# Strengthening post-market COVID-19 vaccine safety surveillance and research: An interpretive description study from Kenya, South Africa, and Canada

**DOI:** 10.1371/journal.pgph.0006142

**Published:** 2026-10-27

**Authors:** Anila Naz AliSher, Shabnam Shaik, Nellie Myburgh, Nomsa Ndaba, Alex Hinga, Winnie Okore, Samuel Sang, Salima Siraj, Thabisile Qwabi, Kimberley Gutu, Allan Matano, Antonia Di Castri, Sonali Kochhar, Eunice Wangeci Kagucia, Ziyaad Dangor, Clare L. Cutland, Karina A. Top, for the International Network of Special Immunization Services (INSIS) Investigators

**Affiliations:** 1 Faculty of Nursing, University of Alberta, Edmonton, Alberta, Canada; 2 South African Medical Research Council, Vaccines and Infectious Diseases Analytics Research Unit, University of the Witwatersrand, Johannesburg, Gauteng, South Africa; 3 Africa Health Research Institute (AHRI), Durban, South Africa; 4 KEMRI - Wellcome Trust Research Programme, Kilifi, Kenya; 5 Wits African Leadership in Vaccinology Expertise (Wits-Alive), School of Pathology, Faculty of Health Science, University of the Witwatersrand, Johannesburg, Gauteng, South Africa; 6 Canadian Center for Vaccinology, IWK Health, Dalhousie University, Halifax, Nova Scotia, Canada; 7 Social and Behavioural Health Sciences Division, Dalla Lana School of Public Health, University of Toronto, Toronto, Ontario, Canada; 8 Department of Global Health, University of Washington, Seattle, Washington, United States of America; 9 Global Healthcare Consulting, New Delhi, India; 10 Department of Pediatrics and School of Public Health, University of Alberta, Edmonton, Alberta, Canada; PLOS: Public Library of Science, UNITED STATES OF AMERICA

## Abstract

Post-market vaccine safety surveillance and research are essential to detect and evaluate rare adverse events following immunization (AEFIs) not identified in pre-licensure clinical trials, such as myocarditis post-COVID-19 vaccination. AEFI surveillance infrastructure varies between jurisdictions. Factors that enable or hinder vaccine safety evaluation across diverse settings are not well understood. The study objective was to examine clinical, social, and structural/system factors that enable AEFI case identification, reporting, data and biosample collection in high-income countries (HICs) and low-and middle-income countries (LMICs). We conducted a qualitative study informed by Interpretive Description in Kenya, South Africa, and Canada. Three participant groups were recruited using purposive and snowball sampling: (1) key informants with leadership roles in AEFI surveillance and research, (2) healthcare workers, research and laboratory staff involved in vaccine safety evaluation, and (3) past participants in COVID-19 vaccine safety studies. Data were collected through semi-structured interviews and focus group discussions. Thematic analysis was conducted. Eighteen key informants, 37 healthcare workers and research staff, and 27 past research participants were enrolled. Facilitators and barriers were identified across two domains: AEFI surveillance and investigation, and vaccine safety research participation. AEFI surveillance and investigation were shaped by trust in system responsiveness, beliefs and awareness around AEFI reporting, digital innovation, and implementation gaps. Research participation was shaped by altruism and social influence, logistics and research infrastructure, and institutional policies and privacy. Facilitators for AEFI surveillance and research included established policies and procedures for surveillance, digital tools, empathic communication about AEFIs, and convenient research processes. Barriers included limitations in workforce capacity, diagnostic testing and funding (especially in LMICs), and cumbersome research approval processes. Findings underscore the need for globally harmonized standards and locally tailored strategies to support AEFI surveillance and research in LMICs and HICs that are patient/participant-centered and include sustainable investments in infrastructure and workforce capacity.

## Introduction

Effective vaccines against COVID-19 were developed with unprecedented speed and efficiency. As with some previous mass vaccine campaigns [1], rare adverse events following immunization (AEFIs) were identified after COVID-19 vaccines were introduced, including thrombosis with thrombocytopenia syndrome (TTS) associated with adenoviral vector COVID-19 vaccines (e.g., ChAdOx1-S, AstraZeneca [Vaxrevia], Ad26.COV-2S, Janssen) and myocarditis and pericarditis associated with mRNA COVID-19 vaccines (i.e., BNT162b2, Pfizer-BioNTech [Comirnaty]; mRNA-1273, Moderna [Spikevax]) [2–10].

The rarity of these AEFIs (occurring in fewer than 1–10 per 100,000 vaccinees) makes them challenging to investigate, as cases present sporadically in different places, times, and after different vaccines. Moreover, the incidence of an AEFI and the background rate of the adverse event in the population may differ by vaccine product, age group, sex, ancestry, geography and other factors, which can complicate detection and confirmation of safety signals [7–11]. The capacity to diagnose and manage patients with AEFIs, as well as accessibility of vaccine and clinical information for surveillance purposes, also differ between health systems and jurisdictions, leading to differences in AEFI reporting rates and health outcomes [7,8,11]. It is therefore essential to have surveillance systems in place to detect and investigate AEFIs in low- and middle-income countries (LMICs) as well as in high-income countries (HICs), to fully characterize the safety profile of a vaccine across different settings and population groups.

However, to truly advance vaccine safety science and reduce the potential for AEFIs, we need to investigate the biological mechanisms and risk factors underpinning AEFIs such as COVID-19 vaccine-associated TTS and myocarditis [12]. This requires the timely, coordinated collection of large numbers of biosamples from post-vaccination cases and vaccinated controls worldwide, using harmonized protocols that enable analysis on common platforms [13]. To date, no single country has been able to collect enough biosamples on its own to conduct highly powered analyses into biomarker risk factors for AEFIs [12].

International networks for vaccine safety research that include LMICs, such as the Global Vaccine Data Network (GVDN) and the International Network for Special Immunization Services (INSIS), have been successfully established, proven feasible, and resulted in valid data generation using harmonized protocols and procedures [5,13–15]. Such networks also hold promise for exploring vaccine safety within populations that may be excluded from pre-licensure clinical trials (e.g., pregnant and breastfeeding individuals, children, older adults, people with [multi-]comorbidities) [15]. The benefits of these networks include capacity-building using standardized and internationally recognized methods and tools; access to information, expert clinical and epidemiological support across diverse sites; and increased statistical power to explore associations between vaccines and rare adverse events [5,13,15]. Access to international network data has also helped researchers identify differences in AEFI reporting rates between countries, informing the interpretation and generalization of findings, and identifying potential gaps in surveillance [11]. There is limited research exploring the successes and challenges of vaccine safety networks in LMICs and HICs, particularly regarding biosample collection in AEFI surveillance, and patient perspectives of experiencing AEFIs and participating in vaccine safety research. The studies with evaluative components have tended to be quantitative [11,15,16] and/or retrospective in focus [11,16,17]. Others offer anecdotal lessons learned [18]. This collaboration among researchers at the Vaccines and Infectious Disease Analytics Research Unit of the University of Witwatersrand (Wits-VIDA) and Wits African Leadership in Vaccinology Expertise (Wits-ALIVE) programme in South Africa, KEMRI-Wellcome Trust Research Program (KWTRP), and Canadian Special Immunization Clinic (SIC) Network via INSIS, presented a unique opportunity to investigate facilitators and barriers to vaccine safety surveillance and research across different population groups and settings.

The study objectives were to use an exploratory, multiple-case study approach to provide detailed qualitative findings about the clinical, social, and structural/system factors that contribute to the success of AEFI surveillance and biosampling of cases and controls across LMIC and HIC contexts, and to characterize factors that facilitate and complicate AEFI surveillance and biosampling.

## Materials and methods

### Ethics statement

Ethical approval for the study was obtained from the University of Alberta Human Research Ethics Committee (Pro00136220), IWK Health Research Ethics Board (Project #1028279), KEMRI Scientific and Ethics Review Unit (SERU; Protocol no. 5133) and from the University of the Witwatersrand’s Human Research Ethics Committee (HREC - Medical) (clearance certificate no. M230777). Written or electronic informed consent was required. To preserve confidentiality, participants were assigned a unique ID based on their participant group (key informant [KI], healthcarework/research staff [HCW/RS], past research participant [PP]), participant number, and study site (Kenya [KE], South Africa [SA], Canada [CA], Canada/International [CA/INT] for international KIs interviewed from Canada).

### Study design and approach

This study employed a qualitative multiple-case study approach informed by Interpretive Description [19] to examine three different contexts (South Africa, Kenya, and Canada), which are geographically bounded and had unique experiences and perspectives on COVID-19 vaccine safety surveillance. Interpretive description was chosen as it supports the integration of multiple knowledge forms (e.g., clinical, experiential, contextual) to generate practical insights. This design was considered most conducive to gaining insight into overarching similarities and key differences between contexts to inform policy and program recommendations for strengthening vaccine safety evaluation globally.

### Study participants and recruitment

Three groups of participants were recruited: (1) key informants (KIs) with expertise in vaccine safety surveillance or policy in leadership positions in governmental organizations and/or academia; (2) healthcare workers and laboratory and research staff (HCW/RS) involved in AEFI surveillance, recruitment, biobanking, or clinical management of AEFI cases; and (3) past participants (PP) in post-market COVID-19 vaccine safety studies. Participants were enrolled at three sites: Wits-VIDA in Johannesburg, South Africa; KWTRP in Kilifi, Kenya; and the University of Alberta in Edmonton, Canada.

We recruited participants using a combination of purposive, convenience, and snowball sampling through various strategies tailored to each site and participant group, including emailed or mailed invitation letters, and phone calls. To address potential referral bias inherent in snowball sampling, efforts were made to purposively seek variation in participants’ professional roles, settings, and prior experiences.

To capture a broad range of views on AEFI surveillance and research in HIC and LMIC contexts, KIs were identified from each country and via researchers’ international networks using purposive sampling based on their involvement in national and/or international immunization policy, vaccine safety surveillance, and vaccine research in HICs and LMICs. Each site recruited KIs from their own country, and additionally, the Canadian site recruited KIs based in other HICs and LMICs to include a broad range of perspectives. Purposive and snowball sampling were used for recruiting healthcare workers and research staff, aiming to ensure broad representation of roles and experience.

Past research participants were recruited from among participants in post-market COVID-19 vaccine safety studies who provided consent to contact for future research. In Canada, participants were recruited from a COVID-19 vaccine safety study involving individuals who had experienced AEFIs and were referred to a special immunization clinic [20]. In South Africa, participants were recruited from hospital-based sentinel surveillance of adverse events of special interest for COVID-19 vaccines, with vaccination status determined after enrollment [15]. In Kenya, participants were recruited from a cohort event monitoring study of COVID-19 vaccine safety conducted in Kilifi, a rural setting [21]. These participants were intentionally included because their firsthand experiences with COVID-19 vaccine safety monitoring and, in some cases, AEFIs, provided rich perspectives of the patient/participant experience of AEFI surveillance and research. Eligible individuals were contacted for study participation using purposive sampling to ensure representation of different age groups and genders.

KIs and PPs were recruited to participate in 30- to 60-minute semi-structured interviews. Healthcare workers and research staff were recruited to participate in 60- to 90-minute focus group discussions, with up to two focus groups conducted per site. Paper-based or electronic informed consent was required prior to participation. Consent was reconfirmed verbally before starting each interview or focus group. Consent forms and interview/discussion guides for KIs, HCW/RS and PPs were available in English and were translated into local languages as applicable for each site. At the Canadian site, all interviews, and focus group discussions were conducted in English. At the South African site, key informant interviews and focus group discussions were conducted in English, and past participant interviews were conducted in English or the participant’s preferred language (e.g., isiXhosa, isiZulu). At the Kenyan site, key informant interviews were conducted in English, while focus group discussions and past participant interviews were conducted in English or Kiswahili depending on the participant’s preference.

### Data collection

Interviews and focus groups were conducted by trained research team members from each respective site (Canada: ANA; South Africa: NM, NN, TQ; Kenya: WO, AH, S Sang, AM). Participants were recruited at the Canadian site from 11 December 2023 to 29 October 2024, at the South African site from 16 February 2024 to 23 May 2024, and at the Kenyan site from 29 April 2025 to 4 July 2025.

Data collection methods were selected based on participant roles and the nature of the information sought. We chose to conduct semi-structured interviews with KIs to elicit in-depth perspectives on system-level policy, governance and strategic challenges, and to facilitate scheduling with busy health leaders. Focus group discussions were used with HCW/RS to explore shared experiences of frontline staff with complementary roles on AEFI surveillance and research teams. Interviews were conducted with PPs to ensure comfort and confidentiality in sharing their personal experiences.

The interview and focus group discussion guides were developed based on a comprehensive literature review, expert consultations, and research team discussions and tailored to each participant group (S1 Text). Interview and focus group discussion topics with KIs and HCW/RS covered experiences with AEFI surveillance and research during the COVID-19 pandemic, including policy and procedures, infrastructure, data collection and biosampling practices, institutional supports and barriers, and inter-institutional collaboration. PPs were asked about experiences with COVID-19 vaccine decision-making, vaccination itself, and participating in a vaccine safety research study.

At the Canadian site, discussion guides for HCW/RS participants were piloted with two healthcare workers with prior involvement in vaccine research, one of whom had personally experienced side effects from a COVID-19 vaccine, to assess the clarity and relevance of the questions. Minor modifications to the guides were made based on their feedback. All sites reviewed the interview and discussion guides for suitability to the local context.

Sample sizes were established in advance to allow for a broad range of experiences and perspectives to be represented across diverse contexts. Interviews were conducted in person or online via cloud-based communication platforms designed for video conferencing and audio recorded. Data collection continued until each site reached its planned sample size for each participant group. In line with narrative and interpretive qualitative methodologies, the emphasis was on capturing rich, diverse accounts rather than pursuing thematic saturation [19]. Demographic information was also collected from participants: gender, country, years of experience and role for KIs and HCW/RS participants, and age and gender for PPs.

### Data analysis

Interview recordings were transcribed, translated (as applicable), and de-identified by one to two research team members at each site. Transcripts were uploaded into NVivo software in Canada (version 12.6.1.970) and Kenya (version 15.2.0) and to Dedoose (version 9.0.107) in South Africa for analysis. Braun and Clarke’s six-phase thematic analysis was chosen because it provides a transparent and adaptable analytic framework that supports the systematic, iterative construction of themes from qualitative data. Consistent with contemporary understandings of thematic analysis, this approach recognizes that themes are actively developed through sustained analytic engagement rather than passively emerging from the data, while maintaining flexibility to align with the study’s epistemological and methodological commitments [22,23]. The six-step process included: (1) familiarization, (2) initial code generation, (3) theme development, (4) theme review and refinement, (5) theme definition, and (6) final write-up. Two to three trained analysts at each site conducted coding of transcripts (Canada: ANA, S Siraj; South Africa: S Shaik, TQ, NN; Kenya: WO, AH, S Sang). Discrepancies were resolved through discussion with the full research team across all three sites during iterative analysis meetings. Code books were reviewed and discussed regularly among the sites to identify similarities and differences.

The analysis was guided by Interpretive Description methodology [19]. Rigour was ensured through strategies aligned with trustworthiness criteria, including credibility, dependability, transferability, and confirmability [23]. Credibility was enhanced through prolonged engagement with the data, beginning with multiple readings of transcripts to build a thorough understanding before detailed coding. Dependability was achieved via a comprehensive audit trail, comprising coding logs, analytic memos, and documentation of methodological choices. Confirmability was improved by using reflexive practices, like keeping reflective field notes after each interview to record assumptions, potential biases, and immediate impressions. Transferability was ensured by offering detailed, contextual descriptions of the study setting, participants, and findings, enabling readers to assess its applicability to other contexts. The study adhered to the Consolidated Criteria for Reporting Qualitative Research (COREQ) to improve methodological transparency and thoroughness (S2 Checklist) [24].

To support cross-site analysis and interpretation, an in-person research team meeting was held in Johannesburg, South Africa, from May 6–8, 2025, bringing together investigators and field researchers from all participating sites. During this meeting, teams shared preliminary findings, discussed emerging themes, and reflected on context-specific challenges and methodological considerations. This collaborative engagement enabled comparative reflection, deepened cross-site interpretation, and contributed to refining the thematic structure used in subsequent analysis. The meeting also facilitated alignment in analytic approaches while maintaining contextual specificity. As part of the broader analytic process, a stakeholder power analysis was conducted to map the influence and roles of key actors, including policymakers, healthcare workers, community leaders, and families, in shaping AEFI surveillance and reporting practices across sites [25,26].

### Inclusivity in global research

Additional information regarding the ethical, cultural, and scientific considerations specific to inclusivity in global research is included as Supporting Information (S1 Checklist).

## Results

### Participant demographics (Table 1)

Across all sites, the team conducted 18 key informant interviews, five focus group discussions involving 37 healthcare workers and research staff, and 27 interviews with past research participants. Most participants were female. KIs reported between 2.5 and over 30 years of experience in their current roles. The HCW/RS group included both clinical and laboratory research staff with experience ranging from 1 to over 20 years. Past research participants ranged in age from 22 to 74 years.

**Table 1:** Demographic Information of all research participants across sites.

Key Informants			
	South Africa	Kenya	Canada/International*
Total Participants	4	5	9
Male	0	2	6
Female	4	3	3
Years of experience in current role	2.5–28 years	10–14 years	4–30 + years
Geographic Diversity	South African	Kenyan	3 based in Canada 3 based in US 2 based in Australia 1 based in Europe
Experience working in LMIC	24–40 years	15–19 years	3 KIs reported 6–30 years experience working in LMICs
**Healthcare worker and research staff participants**			
	South Africa	Kenya	Canada
Total Participants	13	16	8
Male	2	9	0
Female	11	7	8
Years of experience in role	1–20 + years	5–20 years	2–10 + years
Role			
Research staff	6	8	5
Laboratory Staff	7	8	3
**Past research participants**			
	South Africa	Kenya	Canada
Total Participants	13	6	8
Male	4	4	5
Female	9	2	3
Age range	23–58 years	25–74 years	22–59 years

*International participants were key informants based in high-income countries outside Canada; they were interviewed by a researcher based in Canada.

Two overarching themes were developed from the study: (1) AEFI surveillance and investigation across contexts, and (2) vaccine safety research participation and conduct. Each theme has interrelated sub-themes that capture the nuanced dimensions of participants’ experiences and perspectives (Fig 1).

**Fig 1:**
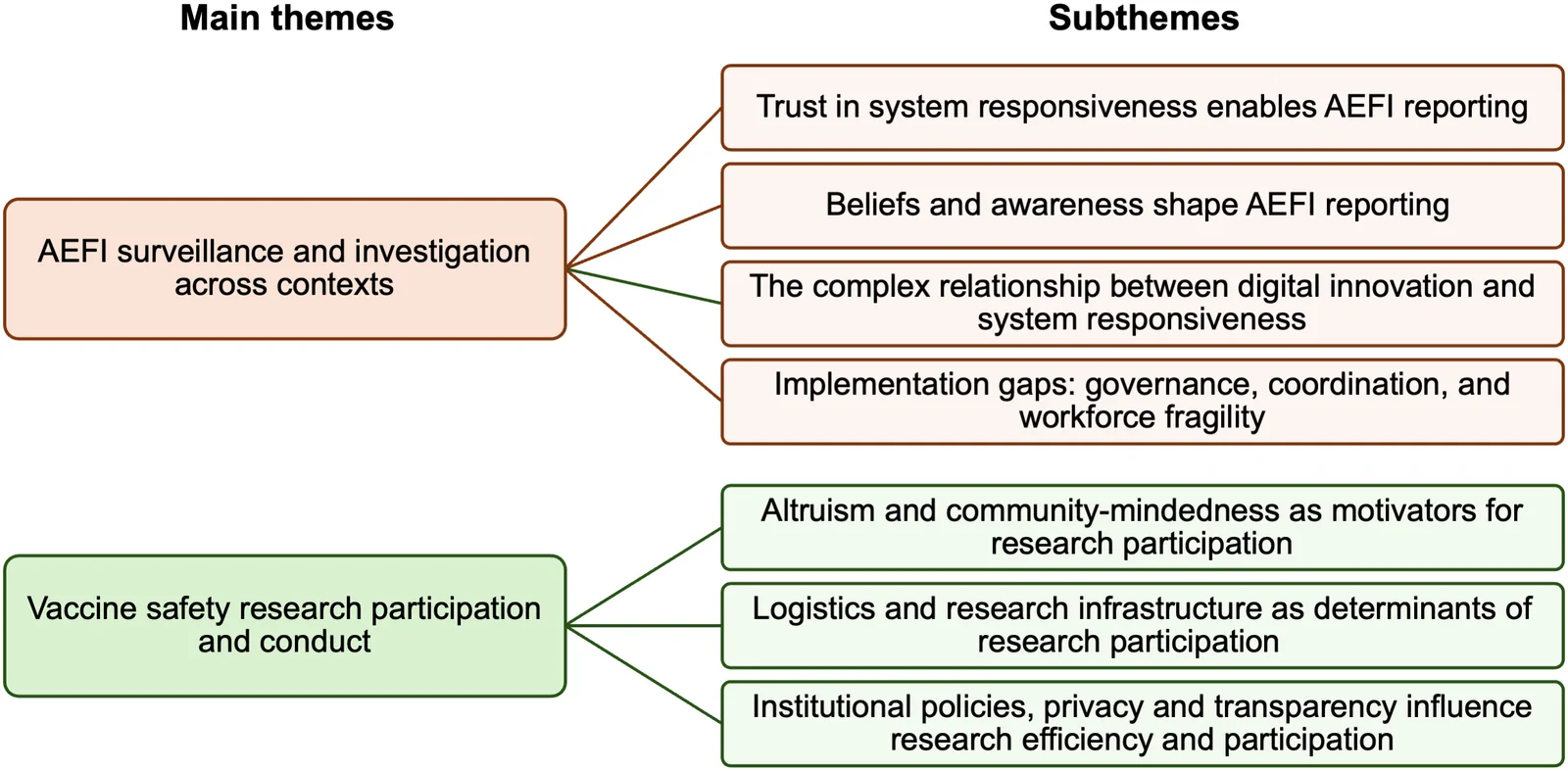
Overview of the major themes and subthemes identified in the study.

### AEFI surveillance and investigation across contexts (Table 2)

This theme explores perceptions related to readiness and responsiveness of public health surveillance and healthcare systems in Canada, Kenya, and South Africa to monitor, investigate, and respond to AEFIs. The multi-country findings reveal both shared priorities and site-specific gaps across four subthemes: 1) trust in system responsiveness enables AEFI reporting, 2) beliefs and awareness shape AEFI reporting, 3) the complex relationship between digital innovation and system responsiveness, and 4) implementation gaps: governance, coordination, and workforce fragility.

**Table 2:** Major theme 1: AEFI surveillance and investigation across contexts subthemes and supporting participant quotes.

Sub-themes	Supporting Quotes
**Trust in system responsiveness enables AEFI reporting**	**Facilitators for AEFI reporting** *“In high-income countries, people expect quick actions when reporting a serious problem. So, they tend to report the adverse events”.* (Key Informant [KI] 2: Canada/International [CA/INT]) “*So, what we do is that the patients who reported side effects were quickly referred for follow-up, ensuring that their cases were managed effectively and that they received appropriate care.”* (Healthcare worker/research staff participant [HCW/RS] 4:CA) “*Vaccine Injury Support Program helps connect individuals to resources when needed, promoting trust in reporting systems.*” (HCW/RS 4:CA) *“They had my phone number, so they would just call to check on how I was doing. Through the questions they asked, they were able to get a better understanding of how the injection was reacting with the human body.”* (Past participant [PP] 3: Kenya [KE]) **Barriers to AEFI reporting** **“***I think there needs to be more belief among healthcare providers. As a healthy 20-year-old female, it took a lot of self-advocacy to make them understand that something was wrong with me. After having myocarditis, I experienced multiple hospital visits with the same set of symptoms, but most of the time, I was met with disbelief. I think there might have been a little bit of gender bias because my symptoms were often dismissed as related to my menstrual cycle or stress. But yeah, I think we need to work on empathy in our healthcare system. I understand that we’re burnt out, it’s a tough field to be in. Still, having belief, showing empathy, and advocating for patients is definitely needed*”. (PP 7:CA)
**Beliefs and awareness shape AEFI reporting**	**Facilitators to AEFI reporting** *“Health education or capacity building [among] the clients was an important aspect in making them come and seek, like report to us that they’ve experienced [adverse events].”* (KI 1:KE) **Barriers to AEFI reporting** *Misinformation… the vaccines were initially quite vilified… very difficult to disentangle what really is related to the vaccines.”* (KI 2:KE) “*Some people with different cultural backgrounds were reluctant to provide any data and relied on home remedies for minor AESIs (sic) [adverse events following immunization]*” (KI 6:CA/INT) *“There’s a lot of alternative medicine… so people won’t say when they went for it… some who had symptoms of AESIs [adverse events of special interest] went for alternative medicine.”* (KI 2:KE) “*Somebody may have died due to an adverse event, and there were no samples done […] you know some people died within a short period of reaching the hospital […], Culturally, the practice there was to have a funeral service and bury the dead within 24 hours. So the ability to do a field investigation or even limited non-invasive autopsies like a whole-body CT scan, was just absolutely not possible. […], It was very difficult to even interview the family because things moved so quickly after death*” (KI 8:CA/INT) *“Religious beliefs... some believe that if you get vaccinated, you really haven’t done the right thing...So, if you get vaccinated and you are in such a religion, then probably you wouldn’t be bold enough to come out and say, I got vaccinated and I’m experiencing this.”* (KI 1:KE) *“Initially vaccines were available only to healthcare providers...The challenge with that is that healthcare providers are a biased population in the sense immediately you feel something, you know what to do about it. So very few of them would have sought care.”* (KI 2: KE) *“There was that hesitancy to report AEFIs. So, it’s not a big deal. Like, you see these people, health workers, would prefer to not even prefer, but from their knowledge base, would basically just encourage somebody, and then they would not report the case. So, the bulk of the cases were not actually being reported, because people just dismiss them offhand, because it’s non-serious, yeah, and then you can go home.”* (KI 3: KE) “*Patients […] don’t understand the importance of reporting [back]*” (KI 4:CA) *“Maybe lack of literacy or education on what to expect or maybe some would confuse them [AEFI symptoms] for other things, other health conditions that they were experiencing.”* (KI 1:KE) *“… a person’s perceptions of seriousness differ … so while medical professionals might say that this is not a serious event, the person experiencing the adverse event would think otherwise … communication between healthcare workers and the community could have been better to improve reporting*” (KI 1:SA)
**The complex relationship between digital innovation and system responsiveness**	**Facilitators for AEFI reporting and investigation** **“***In collaboration with SAHPRA [South African Health Products Regulatory Agency] and MHRA [Medicines and Healthcare Products Regulatory Agency], we introduced an electronic reporting system called the MedSafety App … and not only healthcare workers were able to report adverse events***”** (KI 1:SA). *Another resource was the AU3S [African Union Smart Safety Surveillance], which I think might not have been well publicised, but it was also a platform for capacity building, and experience sharing between countries”* (KI 4:SA) *“Establishing a streamlined process for adverse event reporting helped reduce redundancies and made it easier for healthcare providers to submit necessary data.”* (PP 7:CA) “*We’re lucky that we have the electronic medical record systems in place and we were able to see immunization status, all the immunizations were usually reported through the same mechanism*” (HCW/RS 4:CA) *“So, we have a web-based [platform], which is called the Pharmacovigilance electronic reporting system and then we also have a mobile based solution so you can find the same reporting forms on a mobile application... we also have a (USSD) Unstructured Supplementary Service Data code that they use, then now they’re able to report to us what adverse events they are experiencing*...*it is a versatile enough [with] different types of channels that accommodate reports from the healthcare professionals, but also from general public”* (KI 5: KE) *“Supported causality assessment processes at the national level and the Pharmacy and Poisons Board (PPB) is also part of a regional Information sharing mechanism...It’s a member of the AU-3S. We actually participated in the signal management processes with other four countries under the AUDA-NEPAD [African Union Development Agency-The New Partnership for Africa’s Development] AU-3S. So whatever recommendations that were coming through this program, we were expected to implement it in country.”* (KI 5: KE) **Barriers to AEFI reporting and investigation** “… *the problem with the manufacturers reporting the adverse event is that they report the adverse event anonymised. So that makes it impossible for the National Department of Health to investigate those cases because it might say something like an unknown person in an unknown province received the Johnson vaccine and died 14 days later. We cannot do anything with that information… we can’t match it to our cases… while they [manufacturers] are obliged to report adverse events, the challenge is that if it anonymised, it is not useful for investigation or causality assessment.”* (KI 1:SA) “*Actually, sharing our data is pretty difficult from a government point of view, privacy point of view, and ethics point of view, [which] has been a little bit of a barrier to contributing our data to policy decisions*.” (KI 6:CA) *“Parallel reporting… [name of an online portal] and the regulatory authority system… disintegrated data…because, you’re having it from different sources”* (KI 1: KE) *“I don’t remember getting feedback now from the board [in PvERS (Pharmacovigilance Electronic Reporting System)] on whether this was an adverse event or maybe following up, so how is the patient, can we get their samples?”* (KI 1:KE) *“It would be interesting to know...were they overwhelmed with those data or did they just ignore them...we personally every month put in a lot of data and yeah, the system is not robust.... I think you need a system that’s a little bit responsive...rather than you feeling like you’re throwing things into a black hole.”* (KI 2: KE) “Technology issues...For example, if you are in (place mentioned), or wherever, far-to-reach areas where you don’t have Internet, you know, the *271 (USSD) Unstructured Supplementary Service Data that I gave, that one is just meant for, like, some sort of initial prompt to a nearby facility to be able to report. But even if you go there and you are not able to have them reported, because of Internet issues.” (KI 3:KE) *“Not having an integrated … digitalized record system was a big challenge because you might see someone here and then they go for the same complaint at another facility. There’s no coordination of what happened in another facility. So, we are not electronic, not then, not now.”* (KI 4:KE) *“We didn’t have a lot of systems in place, structures at our level, especially for us as the vaccinators and for us as the people who are at the primary level of interaction with the client.”* (KI 1:KE) *“You have limited capacity for specialized tests.*” (KI 5:KE) “*…the other issue is that in LMICs, there’s no separate fund for treatment of these people*.” (KI 4:CA/INT)
**Implementation gaps: governance, coordination, and workforce fragility**	**Facilitators of AEFI reporting and investigation** “*We followed established [name of organization] guidelines closely to manage and report adverse events, which provided a structured approach during a time of uncertainty.*” (KI 1:CA) *“Then we also have (SOPs) Standard Operating Procedures. Your SOPs need to be clear, if I get an adverse event, what should I do so that you get harmonized treatment of the participants and be able to identify the adverse events.” (*HCW/RS 8:KE) *“We relied heavily on the regional Vaccine Adverse Events Reporting System (VAERS), which allowed us to capture local data and follow up on adverse events as they were reported.”* (KI 2:CA/INT) “*We already have mobile clinics in a lot of provinces, [...] which provide a great platform to report any adverse events” (*HCW/RS 2:CA) *“The investigation teams are drawn from the county health management teams… organized to conduct investigation.”* (KI 5:KE) *“Experts were increased on NISEC [National Immunisation Safety Expert Committee] because predominantly it was paediatricians and so many people who were dealing with childhood vaccinations but we needed to know people who can talk to geriatric care, people who can talk about pregnant women … we needed a whole range of experts”* (KI 4: SA) *“We set up special clinics specifically for those experiencing adverse reactions or those at high risk, which allowed for specialized follow-up and monitoring*.” (KI 5:CA) *“Having a very active National Vaccine Safety Advisory Committee…with scientific evidence…causality assessment.”* (KI 5:KE) **Barriers to AEFI reporting and investigation** *“There was also a challenge with the (MOH) Ministry of Health guidelines regarding how you could detect for the thrombosis and the myocarditis… So, you might find, as the lab department, you just can’t initiate.”* (HCW/RS 8:KE) *There was a confusion over responsibilities at the federal level… overlapping roles, making it unclear who held primary responsibility for various aspects of safety and adverse event monitoring and reporting.* (KI 3:CA) *“Our system is mainly passive… we wait for the patients… we end up missing a lot of them.”* (HCW/RS 1:KE) *“We didn’t have adequate supply of the white forms, the physical ones…because we didn’t have the resources. So, basically, it’s like there’s a serious resource constraint for us to be able to really accomplish the role in 100% of what we would have liked.”* (KI 3: KE) “…*the resources within departments and the various provincial departments didn’t really change. So, to be honest, they were trying to do more work with the same amount of people*” (KI 1:SA) *“I remember doing investigations, using my own resources, yeah, like going all the way to (place mentioned) to follow up someone in their home…my little salary. We don’t have any good support towards this, even from both levels of government. So, we expect the returns to come, but there are no resources to be able to do the particular work.”* (KI 3:KE) “*There is a real misunderstanding among a lot of vaccine providers about what an expected reaction would be, what is serious, what isn’t, what warrants further investigation, and what doesn’t*.” (HCW/RS 4:CA) *“If the healthcare professionals are not aware of what they are supposed to be looking out for… it becomes difficult.”* (KI 5:KE) “*Many individuals don’t have a primary care provider, which limits access to information about AESI*...” (HCW/RS 3: CA) *“So, nobody was talking to these recipients, telling them, if you feel this, this may be associated with the vaccine, so you should come back to the hospital, you need to report. People didn’t even know the reporting channels.”* (HCW/RS 1:KE) “*Primary care centers are overburdened and don’t have enough staff to manage everything*.” (HCW/RS 7: CA) “*Doctors referred many patients to us [at immunization clinics], and then we referred them to public health, creating a backlog*.” (HCW/RS 2:CA) *“The investigation teams of the counties, sub counties sometimes were not, you know, readily available to do the follow up because of competing tasks”* (KI 5:KE) *“I think workload. So, there was a lot of work and we were few. People were off the grid, people who either tested positive or were exposed. You remember, we were telling people, if you had exposure, don’t come. Stay home. If you have suspicion, don’t come. So, we were always having a short number of hands on the ground.”* (KI 4:KE) “*Before COVID there was a lot of training… there’s also a lot of staff turnover. So, the people you train are not necessarily the same ones that are there today. There was a lot of new entrants into the workplace*” (KI 4: SA) *So in low- and middle-income countries, it particularly relates to human resources and healthcare services, and the availability of testing and timing, [...], So for example, we’d hear about a case of possible TTS in one of LMIC we worked and there was very minimal health service there. The patient, I think, recovered and went home. No samples were taken, and they were notified at some point after 10 days. So there’s delay in notification, limited healthcare services, and healthcare staff not familiar with what type of sample or investigation to conduct and why, because they are not necessarily understanding or trained in what particular AESIs might be relevant, and thinking of that in terms of vaccine. So the patient may have already recovered, and it may be too late to get a biologically relevant sample for the condition.* (KI 8:CA/INT) “… *we just didn’t have enough people doing sample collection*” (HCW/RS 5:SA)

CA, Canada; CA/INT, international key informant participant recruited at Canadian site; HCW/RS, healthcare worker/research staff participant; KE, Kenya; KI, key informant; PP, past research participant; SA, South Africa.

#### Trust in system responsiveness enables AEFI reporting.

Across contexts, AEFI reporting was described not simply as a technical surveillance task but as a relational process grounded in trust. Participants’ accounts suggest that willingness to report adverse events depends on whether individuals perceive the system as responsive, accountable, and attentive to their experiences. In this way, reporting was framed as a reciprocal exchange: when institutions demonstrate care through follow-up, referral, and communication, individuals are more likely to view reporting as meaningful and worthwhile.

Participants in Canada emphasized that expectations of timely institutional action shape reporting behaviour. As one key informant stated, in settings where people anticipate “*quick actions when reporting a serious problem*,” (KI 2:CA/INT), they are more inclined to report AEFIs. Participants who were healthcare workers described structured referral pathways and follow-up processes that reinforced this expectation, explaining that individuals reporting side effects were “*quickly referred for follow-up, ensuring that their cases were managed effectively and that they received appropriate care”* (HCW/RS 4:CA). Institutional programmes that support individuals after AEFIs were also described as strengthening confidence in the reporting system by signalling that reports lead to tangible support.

A similar pattern was noted in Kenya, where participants highlighted the importance of follow-up contact after reporting an AEFI. Being contacted by healthcare providers was interpreted as a sign of accountability and concern, reinforcing trust in the system. One PP described how follow-up calls helped them feel that their experience was being taken seriously and that their symptoms were being monitored. These interactions contributed to the perception that reporting was not a one-off task, but part of an ongoing relationship between patients and the healthcare system.

At the same time, participants’ narratives also illustrated how trust can be weakened when individuals feel dismissed or are not believed. For instance, a Canadian PP who experienced myocarditis described repeated encounters in which her symptoms were attributed to stress or her menstrual cycle rather than investigated as a potential vaccine-related adverse event. Her account underscores the importance of empathy and credibility in provider-patient interactions, highlighting how gender bias and clinician dismissal of symptoms can undermine confidence in the system’s responsiveness.

Overall, these findings suggest that trust functions as a key mechanism linking individual experiences to surveillance systems. Follow-up, empathy, and visible institutional action signal that reports are valued and that the system is responsive.

#### Beliefs and awareness shape AEFI reporting.

Across sites, KIs shared that AEFI reporting is shaped by how individuals understand, interpret, and frame post-vaccination symptoms. These key informants reflected on patterns they observed in communities and clinical settings, noting that adverse events were often normalized as expected reactions, dismissed as minor, or confused with unrelated health conditions. When symptoms were perceived as routine or not serious, they were less likely to be reported through formal surveillance channels. In this way, reporting was influenced not only by awareness of systems but also by how individuals judged whether their experience was important enough to warrant action.

Beliefs and health practices also shaped decisions about seeking healthcare and reporting AEFIs. Individuals were sometimes said to rely on “*home remedies*” (KI 6:CA/INT) or turn to “*alternative medicine*” (KI 2:KE) to manage post-vaccination symptoms. This suggests that adverse events might be managed within informal systems of care and thus go undetected even when surveillance mechanisms are available.

Social and religious norms were also described as influencing individuals’ willingness to disclose vaccination-related symptoms. In Kenya, KIs noted that in some religious contexts, accepting vaccination was viewed negatively, making it socially difficult to admit experiencing a vaccine-related health issue. As one KI explained, *“Religious beliefs… probably you wouldn’t be bold enough to come out and say, I got vaccinated and I’m experiencing this”* (KI 1:KE). In such contexts, AEFI reporting was not only a health decision but also a socially sensitive act shaped by community expectations and potential stigma. Similar concerns about stigma and reluctance to disclose AEFIs were echoed by KIs recruited from Canada with experience in LMICs, who observed that social perceptions and personal beliefs could discourage open reporting of symptoms.

Together, these findings show that awareness of reporting systems alone is not enough to ensure that AEFIs are reported. Even when people know that reporting is possible, they may not do so if symptoms are seen as normal, attributed to other illnesses, considered too minor, or deemed socially risky to disclose.

#### The complex relationship between digital innovation and system responsiveness.

Across Canada, Kenya, and South Africa, participants described digital technologies as increasingly central to AEFI reporting, suggesting a shift toward more streamlined and accessible surveillance processes. South Africa implemented digital tools such as the MedSafety App, which enabled AEFI reporting by patients and healthcare providers, while data-sharing via the African Union Smart Safety Surveillance (AU-3S) system enabled regional collaboration and comparison of reporting rates between countries. Similarly, in Kenya a digital tool referred to as the Pharmacovigilance Electronic Reporting System (PvERS) facilitated reporting as well as data-sharing via the AU-3S, enhancing regional collaboration. Participants from Canada also reported that streamlined digital reporting systems enhanced AEFI reporting infrastructure.

However, participants’ accounts also revealed that technological solutions did not automatically translate into improved system responsiveness. In Kenya, limited feedback following submissions through PvERS contributed to a perception that reports disappeared into the system, with one participant describing the experience as “*throwing things into a black hole*.” (KI 2:KE). This absence of feedback weakened the perceived value of reporting and undermined engagement with surveillance processes. In both Kenya and South Africa, participants further described limited integration of electronic databases, incomplete vaccine record systems, and restricted access to diagnostic testing, which constrained the ability to validate adverse events and weakened confidence in reporting outcomes.

Data governance and information-sharing challenges further complicated coordination and hampered responsiveness. In South Africa, anonymized manufacturer reports were described as protecting privacy but limiting the ability of national authorities to conduct follow-up investigations or causality assessments. In Canada, stringent privacy legislation and institutional data-sharing restrictions were perceived as slowing collaboration and the timely use of surveillance data. In Kenya, parallel reporting platforms and fragmented data systems were described as producing disjointed information flows, reducing the coherence of surveillance datasets. Across contexts, these findings suggest that data protection and system fragmentation can inadvertently limit the practical usability of surveillance information.

Participants highlighted that digital tools were embedded within broader structural conditions that shaped their sustainability and effectiveness. While technology can enhance the visibility and flow of AEFI reports, the value of AEFI surveillance depends on ensuring that reports consistently lead to timely investigation, follow-up, and action.

#### Implementation gaps: Governance, coordination, and workforce fragility.

Across sites, KIs and HCW/RS participants acknowledged that policies and procedures for AEFI surveillance were generally well-established and aligned with national and global guidance. However, their accounts highlighted persistent challenges in how these frameworks were implemented, coordinated, and supported in practice. While formal guidelines, expert committees, and reporting pathways were described in Canada, Kenya, and South Africa, participants indicated that gaps in coordination, clarity of roles, and system capacity often limited the ability to translate policy into timely investigation and response.

In Canada, structured institutional guidelines, specialist referral pathways, and support programs were seen as strengthening the formal architecture of surveillance. However, participants described uncertainty regarding the division of responsibilities among federal agencies involved in vaccine safety oversight. While healthcare delivery and case investigation clearly fall under provincial and territorial jurisdiction, ambiguity at the federal level about leadership and coordination of surveillance functions was perceived to slow communication and decision-making. This suggests that even within well-established systems, strong policy frameworks do not always translate into clear operational leadership.

In Kenya and South Africa, national guidelines and expert review mechanisms were also described, including regional investigation teams in Kenya and the expansion of national and provincial immunization safety expert committees in South Africa. Yet participants noted that the absence of clear diagnostic pathways for specific AEFIs, such as myocarditis, and the reliance on passive reporting systems limited case detection and follow-up. Where systems depend primarily on patients presenting to care, participants felt that AEFIs were often missed, indicating that surveillance sensitivity is tied to how actively systems seek and verify cases.

Across all settings, workforce limitations emerged as a central constraint linking policy intentions to operational realities. Participants described health systems as overextended, with staff managing multiple responsibilities alongside AEFI reporting and investigation. In Canada, this manifested as heavy care burdens within primary care, where limited time and staffing restricted capacity to support AEFI reporting and follow-up. In LMIC settings, workforce shortages were described as more structural, including insufficient staffing for investigation, limited capacity for sample collection, and high turnover that disrupted continuity and eroded the impact of training. Pandemic-related pressures further intensified these strains, with systems “*trying to do more work with the same amount of people*” (KI 1:SA). Together, these conditions contributed to backlogs, delayed investigations, and reduced system responsiveness.

Overall, participants’ accounts indicate that AEFI surveillance systems are most vulnerable at the level of implementation. Policies, committees, and reporting structures may exist, but gaps in coordination, education, workforce stability, and diagnostic capacity constrain the system’s ability to act on AEFI reports. Surveillance effectiveness, therefore, depends not only on formal governance arrangements but on sustained investment in human resources, training, and clear accountability pathways that enable timely investigation and response.

### Vaccine safety research participation and conduct (Table 3)

This theme captures participants’ views and experiences related to receipt of COVID-19 vaccination and participation in vaccine safety research. Participants across all three sites recognized vaccine safety research as important for strengthening public confidence and informing immunization programs. However, engagement in research was shaped by a combination of motivational factors, logistical feasibility, institutional research processes, and trust in how data and findings are managed. Three key subthemes were identified: (1) altruism and social influence as motivators for research participation, (2) logistics and research infrastructure as determinants of research participation, and (3) institutional policies, privacy and transparency influence research efficiency and participation.

**Table 3:** Major Theme 2: Vaccine safety research participation and conduct subthemes and supporting participant quotes.

Sub-themes	Supporting Quotes
**Altruism and social influence as motivators for research participation**	*“I know how important it is… I’m here today because of others’ trial studies and stuff 30 years ago”* (PP1: CA) *“Since the study was looking for genetic markers, I thought maybe I could serve as a kind of baseline or historical reference — like, this happened before, is there something in common? That made me more willing to participate, knowing that my history and my health could actually help others* (PP1: CA) *“The way I felt was that I wanted to be part of the study because it’s something that would help me personally…If there’s another study that will be ongoing, I can just participate and continue with it.” (*PP1: KE) *“I have absolutely no problem, I wouldn’t have any bad feelings because it’s just a sample and we would be continuing with this organization.” (*PP1: KE) *“Just take a small amount. I am okay as long as I know how I’m progressing, how my health is. Things about my health, I like them. I’m okay even with blood.”* (PP2: KE) “*I wanted to protect myself, that’s the idea they planted in us that when you’re vaccinated, you’re safe and it won’t be easy to get COVID-19 or spread it*” (PP10: SA) *“It’s that when you look at how COVID was affecting people, and then there was the opportunity to get the vaccine easily to protect yourself from the virus, I just decided to take it without much trouble. I didn’t see any difficulty at all.” (*PP4: KE)
**Logistics and research infrastructure as determinants of research participation**	**Facilitators for vaccination and research participation:** “*It is easy to access [vaccination sites], you don’t have to take a taxi*” (PP9: SA) *“The location where I got the vaccine it was nearby…so I didn’t [need] fare to go far. I got all the doses in a place that was near.”* (PP2: KE) *“The government instituted… [name of an online] system. It enabled us to track the vaccines.”* (HCW/RS1: KE) *“The good one I can think of is the ease of access to providing samples… it was extremely painless.”* (PP3: CA). “*What made it easy was that they used to call, and I wasn’t going anywhere*...*they’d also get the feedback they expected from me quickly. You know sometimes you don’t have your phone with you, you’re out. They call and it rings but then stops, and the caller doesn’t get the information they want from you. But I became more responsive in giving them feedback.”* (PP4: KE) *“The reimbursement for parking and things like that are really important*”(PP7: CA). “*Ensuring we collect the right data early in the study helps avoid gaps... having strong procedures for reconciliation and data cleaning ensures findings are accurate.”* (HCW/RS7: CA) “*We’re fortunate to have access to [hospital] labs, which provide extensive support for PCRs, immunoglobulins...”* (HCW/RS4: CA) **Barrier to vaccination and research participation:** “*There isn’t enough space for people to sit, it’s always packed with grandmothers and young children, and everyone is in the same space. And time also, they don’t respect the time at clinics, we understand that they need to go for lunch but they also need staff so that when others got for lunch, others can still cover for the patients*” (PP4: SA) “*The fact that you go to the clinic in the morning and wait. After waiting, no ones comes to you and gives you some counselling. When you go for an HIV test, you start with counselling first; why wasn’t that done with COVID*?” (PP4: SA) “*I think on our side, the handover process, there are a lot of technicians that left, even the supervisor was not there at some point. So, our training was a little bit offish, so, there was no proper handover of skills and work” (*HCW/RS3: SA)
**Institutional policies, privacy and transparency influence research efficiency and participation**	**Facilitator to vaccination and research:** *“Well, after the explanation, that’s what convinced me to get the vaccine. If you explain properly the purpose and benefits of this, then it is easy to accept.” (*PP6: KE) *“I have no problem. If it’s about my health, I have no problem that kind of research is meant to look into such things, so I wouldn’t have any issue with it.”* (PP1: KE) **Barriers to vaccination and research:** *“It is alright, it is all just as I have said you just take your data and tell me we have taken your blood sample of this and that. Now the problem with you or with us is that you take someone’s information and you will not tell them the results.”* (PP6: KE) “*Consent forms can be challenging for participants to understand, particularly in genetics and epigenetics studies.”* (KI4: CA) “*Participants hesitated regarding the long-term use of their genetic data... feared information might be used beyond the initial purpose.”* (HCW/RS2: CA) “*When it comes to providing health information. I think that would kind of raise my brows. Like with saliva, I’m fine, no problem. But with health information, I’d want to know what it’s for, how personal it gets, and whether it could identify me. That’s a big thing for me (*PP4: CA) “*Since follow-up wasn’t legislated, it required ethics approvals and research agreements, which took a long time to process*.” (KI6: CA) *“So, I think if you get your local approvals, it is very important, because if the sub-county has not approved anything... the county health management team doesn’t approve whatever study you want to do, the sub-county will not act and it will trickle down to the hospital...So, if those approvals are not given, even the staff who is working under the in charge will not be able to do anything... as opposed to when you just show up, okay, maybe you only have [an] approval from one area but then did not engage up to down there.”* (HCW/RS 3: KE) *“Another issue whenever we wanted to publish anything, we had lots of barriers. So if we wanted to give, I know, if we wanted to put anything in writing and have it peer reviewed … it took just months and months and months for there to be an ethics approval within our current system. We just weren’t able to deal with that in a timely manner*.” (KI6: CA)

CA, Canada; CA/INT, international key informant recruited at Canadian site; HCW/RS, healthcare worker/research staff participant; KE, Kenya; KI, Key Informant; PP, Past research Participant; SA, South Africa.

#### Altruism and community-mindedness as motivators for research participation.

Participants from both Canada and Kenya expressed strong motivations to participate in vaccine safety research, driven by a desire to help others and contribute to broader scientific and public health efforts. In Canada, this sense of altruism was often linked to contributing to scientific knowledge, particularly genomic and longitudinal studies that participants believed could benefit future generations. Similarly, Kenyan participants viewed participation positively, describing both personal and communal benefits. Some participants explained that continued involvement in research could provide indirect long-term advantages, such as access to health information, medical follow-up, or ongoing relationships with trusted healthcare and research institutions. These “trusted organizations” included local hospitals, university research programs, and public health agencies that participants perceived as credible, community-oriented, and committed to improving health outcomes. South African participants similarly described gaining knowledge about their illness and receiving clearer information about their health as important motivators for participation.

Perspectives from past participants at all three sites reflected how motivations for research participation were shaped by beliefs about protection, responsibility, and collective well-being. Participants described involvement in vaccine safety studies as a way to contribute to broader community protection and to support public health efforts during a time when COVID-19 was severely affecting their communities. Rather than focusing solely on personal benefit, many expressed a sense of shared responsibility and viewed participation as a meaningful contribution to efforts aimed at improving health outcomes for others. In South Africa, participants also highlighted helping others and advancing medical knowledge as important motivators for joining vaccine safety research. Together, these accounts suggest that motivations for research involvement extended beyond individual gain and were grounded in a broader sense of social responsibility, collective protection, and contribution to public health.

#### Logistics and research infrastructure as determinants of research participation.

Logistical factors and research infrastructure played an important role in facilitating research participation in all three contexts. Canadian participants highlighted facilitators such as convenient sample collection processes (providing saliva samples for genomic analysis) and access to hospital laboratories as enabling efficient collection and processing of patient samples. The South African site also noted access to research clinic space and laboratory supported trial conduct. At all three sites, financial compensation and reimbursement for expenses were seen as important enablers for study participants and were appreciated as recognition of the time and effort invested.

Past research participants from South Africa and Kenya also emphasized the importance of physical accessibility of vaccination and research-related services. The proximity and ease of access to vaccination and study sites reduced transportation costs and logistical burden, making both COVID-19 vaccination and research participation more convenient. Conversely, clinic environments that were overcrowded, lacked adequate space, and involved long waiting times without clear communication were described as affecting perceptions of care and the overall acceptability of vaccination services.

Participants further described the role of digital and administrative systems in supporting coordination of vaccination services and research activities. In Kenya, a government-supported online system (Chanjo) was mentioned as enabling tracking of vaccinations and supporting the organisation of vaccine-related services. Standardized data collection and cleaning procedures were noted across settings as important for maintaining research quality. However, logistical barriers were also described. In South Africa, turnover of laboratory staff without adequate handover of responsibilities was reported as disrupting continuity and posing challenges for maintaining consistent research operations.

#### Institutional policies, privacy and transparency influence research efficiency and participation.

Concerns related to privacy, consent, and transparency emerged most strongly among Canadian participants, particularly among KIs and HCW/RS involved in vaccine safety research. They noted that institutional research policies and procedures can affect both the efficiency of research processes and the research participant experience.

In Kenya and South Africa, participants’ experiences with health services also highlighted how institutional practices shaped trust, understanding, and willingness to accept vaccination and engage in research. Kenyan participants described positive encounters where clear explanations from healthcare workers influenced their decision to accept vaccination, noting that when the purpose and benefits of vaccination were explained, it became “*easy to accept*” (PP 6:KE). In contrast, in South Africa, participants compared COVID-19 vaccination experience with other health services (e.g., HIV testing) where individual counselling was routinely provided, suggesting that the absence of individual counselling for COVID-19 vaccination left some feeling insufficiently informed.

Research staff further highlighted the complexity of study consent forms, particularly in specialized areas such as genetic testing, as a barrier to comprehension that could deter participation. Similarly, PPs expressed concerns about sharing sensitive health data when they were unclear about how their information would be stored, used, and protected. Across accounts, transparency, especially regarding future data use and privacy protections, was described as an important consideration influencing decisions to participate in research.

### Integration of stakeholder power analysis

A stakeholder power analysis from this research underscored the importance of harnessing the influence of diverse actors, including politicians, healthcare workers, community leaders, and families to support vaccine communication and AEFI response. Each group holds different forms of decision-making or social capital that can support AEFI response if properly engaged. While institutional actors implement protocols and approve safety decisions, frontline workers build relational trust through direct care. Religious and community leaders, with their emotional and cultural connections, play key roles in information dissemination. Further efforts are needed to engage underserved communities around vaccination, AEFI surveillance and research, using communication channels and tools adapted to the cultures, languages, and education level in those contexts.

## Discussion

This study examined the clinical, social, and structural facilitators and barriers to AEFI surveillance and research conduct and participation during the COVID-19 pandemic in Canada, Kenya, and South Africa. By engaging key informants, healthcare workers and research staff, as well as past participants in post-market vaccine safety research across different health system contexts, this study offers critical insights into multisectoral capacities for vaccine safety monitoring and evaluation in high-income and middle-income settings. Our findings highlight several notable facilitators to effective AEFI surveillance and research, as well as several interrelated barriers. Facilitators included robust policies and procedures for AEFI reporting, investigation and follow up, digital tools, and empathic communication about vaccine safety, as well as convenient research processes. Barriers included limited trained workforce capacity, weak or fragmented surveillance infrastructure, underfunded systems reliant on external donors, communication gaps in public education, diagnostic constraints for confirming AEFIs, as well as cumbersome research approvals and inadequately addressed privacy concerns.

### AEFI surveillance and investigation across contexts

This study highlights the multifaceted nature of post-market vaccine safety monitoring and evaluation, revealing contrasts in preparedness, system maturity, and contextual needs across settings. In high-income contexts, facilitators for robust AEFI surveillance included stakeholder support, regional reporting structures, and consistent communication and training for healthcare providers, as well as specialized clinics for patients experiencing AEFIs. These systems were underpinned by digital platforms, electronic medical records, and established pharmacovigilance infrastructure, which in our study, and in prior research, have been identified as enabling timely safety signal detection and response [27]. Conversely, in this study, and in prior research [28], lack of oversight and accountability for pharmacovigilance, limited awareness of AEFI surveillance processes, and insufficient training among healthcare workers, were seen as impeding reporting and investigation activities, particularly in LMIC settings. These findings are consistent with prior studies from HIC and LMIC settings that demonstrated that integrated reporting systems, interoperable data platforms, and strengthened pharmacovigilance networks can improve the timeliness and completeness of AEFI detection, investigation, and regulatory action [15–18,27,29]. Our findings from South Africa are consistent with the work of Sankar and colleagues, who formally evaluated the South African AEFI surveillance system [16]. Their work also highlighted the usefulness of the digital AEFI reporting application (MedSafety App) in facilitating reporting and noted its lack of integration with the existing AEFI surveillance database as a limitation. They identified regional variation in AEFI reporting rates and capacity to review AEFIs, underscoring the need for resources to increase staffing, improve training, and ensure sustained funding. Findings from Kenya identified similar needs for increased funding and resources for AEFI surveillance, as well as the need for clearer guidance and improved access to diagnostic testing to confirm AEFI diagnoses. These findings echo those of a study of the Nigerian AEFI reporting system, which additionally identified security concerns and fear of being blamed for AEFIs as impeding reporting [28]. Together these findings underscore the importance of sustained investments in workforce capacity and training, and digital reporting systems to strengthen pharmacovigilance, particularly in resource-constrained settings.

The AU3S and active COVID-19 vaccine safety surveillance (ACVASS) study, both of which included South Africa and Kenya, offer examples of how coordination of resources and dedicated support can enhance AEFI surveillance and signal detection capacity in LMICs, including for rare adverse events of special interest [15–17,30]. Both programs enabled integration of AEFI data and strengthening of signal detection capacity, though limited access to digital records, and in the case of ACVASS, a requirement for patient consent to access data, presented challenges to complete case identification [15–17,30].

Across all settings, there is a clear need for integrated communication strategies and tools including digital applications and portals, online resources, training modules, and targeted media campaigns to support public and provider understanding of AEFIs and AEFI reporting. Effective messaging must not only clarify what constitutes an AEFI but also strike a balance between presenting benefits and risks of vaccination, avoiding stigma while addressing safety concerns [28,31–33]. Stakeholders identified key targets for communication efforts, including youth, healthcare workers and vaccinators, as well as community members, and emphasized the importance of leveraging traditional media, social media, and local leaders to build trust and promote evidence-based health information. This resonates with broader recommendations from LMIC and HIC settings to develop localized, multilingual vaccine information campaigns that incorporate trusted community channels and address gaps in health literacy [28,34–39]. Together, these findings highlight that effective AEFI surveillance depends not only on technical infrastructure but also on sustained investments in communication, workforce development, and public engagement.

Moreover, managing surveillance in a rapidly evolving global health environment requires adapting to shifts in international support, such as the contraction of budgets for foreign aid, World Health Organization (WHO) and GAVI, which have direct implications for vaccine procurement and safety monitoring. Strengthening country-level systems and ensuring sustainable investments in pharmacovigilance infrastructure are critical to maintaining public confidence as new vaccines, such as respiratory syncytial virus (RSV) vaccines, are introduced across the life course [31,32].

### Vaccine safety research participation and conduct

Altruism and community motivation emerged as powerful drivers for both vaccine acceptance and research participation in all three settings, although participant concerns and experiences differed across sites. This is consistent with other research showing that individuals often enroll in vaccine trials out of a sense of civic duty and a desire to restore societal normalcy during public health crises [40–42].

Our findings also revealed that fair compensation and reimbursement for study-related burdens are key to participants’ enrollment and retention in research studies. As reported in other studies, addressing these needs promotes equity, respects participant contributions, and supports diverse recruitment [40,41].

KI and HCW/RS participants in all three contexts highlighted research governance and ethics review processes as key challenges to research study initiation and knowledge dissemination. Specifically, delays in obtaining ethics and institutional approvals were described as hindering timely study initiation and follow-up of participants experiencing AEFIs. These procedural delays were also reported to complicate the dissemination of research findings. Our experience is echoed in other studies from LMIC and HIC settings that have similarly reported that lengthy ethics approval processes delayed study initiation and even prevented sites from participating in vaccine safety research [5,15,41]. There is a critical need to streamline and harmonize research ethics and institutional approval processes to enable research that is timely and responsive to public health needs. Agreements for reciprocal ethics approvals between review boards could lessen the burden and cost on both research teams and ethics review committees. Development and implementation of standard operating procedures aimed at streamlining procedural timelines and ensuring that research adheres to international standards of data governance, transparency, and reproducibility could support faster initiation of high-quality multi-centre and multi-national studies, particularly in research contexts where institutional capacity may be a challenge [40–43].

Interestingly, research consent forms were identified by both research staff and past participants as not meeting participant needs. On the one hand, the forms were reported to be complex and difficult for participants to understand, while on the other, participants expressed concerns regarding how their personal health information would be stored, used, and protected. While participants’ information needs likely vary between individuals and by study type, these findings suggest a need for simpler consent forms that better address participants’ information needs.

These insights underscore the need for streamlined institutional protocols, clearer consent processes, and robust data governance to foster trust and support ethical, participant-centred research practices. The WHO Pandemic Agreement provides a roadmap for addressing many of these challenges by calling on member states to promote research collaboration, build research capacity around the world, and support rapid sharing and dissemination of research protocols, materials, and findings [43]. Our findings provide additional insights to support operationalizing the recommendations in the WHO Pandemic Agreement.

### Recommendations (Table 4)

Based on the findings, we developed recommendations for addressing barriers to AEFI surveillance and research that fall into five broad categories: addressing human resource gaps, enhancing surveillance infrastructure, sustained funding, improving communication with the public and underserved groups, and improving diagnostic capacity. Specific strategies include targeted training for frontline healthcare workers, establishment of dedicated AEFI surveillance units, investment in integrated digital AEFI reporting systems including mobile applications that may be more acceptable to younger individuals and improve access in rural areas, sustained national financing, culturally appropriate multilingual awareness campaigns delivered through trusted community channels, and development of standardized investigation protocols and clinical response teams to improve assessment of people with AEFIs.

**Table 4:** Recommendations for addressing barriers to AEFI surveillance and research.

Barrier Category (Resources)	Description	Recommendations
1. Human Resource Gaps	Shortage of trained staff; weak pharmacovigilance teams	Train frontline workers on AEFI recognition and reporting Establish dedicated pharmacovigilance units
2. Surveillance Infrastructure	Weak or fragmented health information systems Lack of knowledge; lack of digital tools	Invest in integrated digital reporting systems Support mobile-based tools for data entry
3. Financial Limitations	Underfunded systems; dependence on external donors	Allocate national budget lines for pharmacovigilance Build sustainable funding partnerships
4. Communication Gaps	Poor media reach concerning AEFI; lack of culturally appropriate materials	Develop localized, multilingual AEFI awareness campaigns Use trusted community channels (radio, leaders)
5. Diagnostic Constraints	Limited lab access; delays in confirming AEFIs	Strengthen referral labs and point-of-care diagnostics Create AEFI investigation protocols with mobile teams

AEFI, adverse event following immunization.

While these recommendations reflect globally relevant principles for strengthening AEFI surveillance systems, their implementation should be tailored to local health-system structures, resource availability, cultural contexts, and regulatory environments. For example, locally tailored strategies to enhance surveillance infrastructure and address human resource gaps may include establishing dedicated local AEFI surveillance units that support development of training modules for local healthcare providers, investment in digital reporting tools appropriate to local technological capacity, and development of clinical investigation protocols that align with local healthcare capacity. Addressing communication gaps may include partnering with local community leaders to deliver culturally and linguistically appropriate awareness campaigns. Locally tailored strategies are also needed to address context specific challenges such as physical security of healthcare teams and patients accessing health services [28].

As revealed in the stakeholder power analysis, effective AEFI surveillance depends on engaging actors who hold different forms of influence, including formal decision-making authority, technical expertise, community leadership, and cultural trust. These findings suggest that strengthening vaccine safety surveillance requires coordinated action across health, regulatory, and community sectors while allowing flexibility for local adaptation.

### Strengths and limitations

A major strength of this study is its multi-country approach, enabling comparative insights across varied socio-economic and health system settings. Qualitative interviews with key informants, frontline health workers and research staff captured detailed, firsthand perspectives from those directly involved in vaccine safety surveillance, research, and patient care, while incorporating voices of research participants provided additional important insights on improving the patient and participant experience.

Certain limitations are acknowledged. The study used purposive sampling, which may limit the extent to which the results can be applied to regions not represented in each country. Although we implemented strategies to gather diverse perspectives, including conducting past participant interviews in local languages and enrolling participants from a rural setting, certain groups, such as youth, non-English speaking or rural populations, might have been underrepresented. In addition, past participants’ views of vaccination, AEFIs, and research may differ from the general population or others experiencing AEFI because of their prior engagement in research. Moreover, since the study was conducted shortly after the COVID-19 pandemic, the findings may reflect the specific urgency and funding patterns of a global health crisis, which may differ from non-pandemic times. Finally, this study did not specifically examine processes of causality assessment, AEFI data analysis, or regulatory action. Future research should explore these processes in different contexts to strengthen understanding of vaccine safety surveillance systems and support evidence-informed responses to safety signals across diverse settings.

## Conclusion

This study sheds light on the differing capacities and challenges in AEFI surveillance and vaccine safety research across three distinct health system contexts. While middle-income countries like Kenya and South Africa continue to face critical barriers related to resources, infrastructure, communication, and community engagement, HICs like Canada, despite having more established pharmacovigilance systems, also faced barriers to AEFI surveillance in those areas, which were more pronounced during a public health emergency such as the COVID-19 pandemic. Cross-cutting themes such as the importance of training, digital infrastructure, trust-building, and coordinated stakeholder roles emerged as foundational to effective vaccine safety monitoring and evaluation. As countries adapt to a changing global vaccine landscape, including new vaccine technologies and shifting funding patterns, there is an urgent need to develop locally embedded, ethically grounded, and systemically supported models for AEFI surveillance, investigation and research. These models must prioritize accessibility, equity, and sustainability to build public trust and safeguard vaccine confidence in future immunization efforts.

## Supporting information

S1 Text Interview guide for key informants, focus group discussion guide for healthcare provider and research staff participants, and interview guide for past research participants. (DOCX)

S1 Checklist Inclusivity in Global Research Questionnaire. Completed PLOS questionnaire on ethical, cultural and scientific considerations related to inclusivity in global research.
(DOCX)

S2 Checklist Completed COREQ checklist. (DOCX)
